# A Feature-Augmented Explainable Artificial Intelligence Model for Diagnosing Alzheimer’s Disease from Multimodal Clinical and Neuroimaging Data

**DOI:** 10.3390/diagnostics15162060

**Published:** 2025-08-17

**Authors:** Fatima Hasan Al-bakri, Wan Mohd Yaakob Wan Bejuri, Mohamed Nasser Al-Andoli, Raja Rina Raja Ikram, Hui Min Khor, Yus Sholva, Umi Kalsom Ariffin, Noorayisahbe Mohd Yaacob, Zuraida Abal Abas, Zaheera Zainal Abidin, Siti Azirah Asmai, Asmala Ahmad, Ahmad Fadzli Nizam Abdul Rahman, Hidayah Rahmalan, Md Fahmi Abd Samad

**Affiliations:** 1 Faculty of Information and Communication Technology, Universiti Teknikal Malaysia Melaka, Melaka 76100, Malaysia; forignn@gmail.com (F.H.A.-b.); raja.rina@utem.edu.my (R.R.R.I.); asmala@utem.edu.my (A.A.); fadzli@utem.edu.my (A.F.N.A.R.); hidayah@utem.edu.my (H.R.); 2 Faculty of Artificial Intelligence and Cyber Security, Universiti Teknikal Malaysia Melaka, Melaka 76100, Malaysia; zuraidaa@utem.edu.my (Z.A.A.); zaheera@utem.edu.my (Z.Z.A.); azirah@utem.edu.my (S.A.A.); 3 Faculty of Computing Informatics, Multimedia University, Cyberjaya 63100, Malaysia; 4 Department of Medicine, Faculty of Medicine, University of Malaya, Kuala Lumpur 50603, Malaysia; hmkhor@um.edu.my; 5 Fakultas Teknik, Universitas Tanjungpura, Pontianak 78124, Indonesia; sholvariza@untan.ac.id; 6 Lee Kong Chian Faculty of Engineering and Science, Universiti Tunku Abdul Rahman, Kajang 43200, Malaysia; madamumikalsom2025@gmail.com; 7 Centre for Software Technology and Management, Universiti Kebangsaan Malaysia (UKM), Bangi 43600, Malaysia; noorayisah@ukm.edu.my; 8 Faculty of Mechanical Technology and Engineering, Universiti Teknikal Malaysia Melaka, Melaka 75450, Malaysia; mdfahmi@utem.edu.my

**Keywords:** Alzheimer’s disease, artificial intelligence, explainable AI, ensemble learning, meta-model, clinical data, MRI, MMSE, lateral ventricles, CNN, clinical decision, SHAP, Grad-CAM

## Abstract

**Background/Objectives:** This study presents a survey-based evaluation of an explainable AI (Feature-Augmented) approach, which was designed to support the diagnosis of Alzheimer’s disease (AD) by integrating clinical data, MMSE scores, and MRI scans. The approach combines rule-based reasoning and example-based visualization to improve the explainability of AI-generated decisions. **Methods:** Five doctors participated in the survey: two with 6 to 10 years of experience and three with more than 10 years of experience in the medical field and expertise in AD. The participants evaluated different AI outputs, including clinical feature-based interpretations, MRI-based visual heat maps, and a combined interpretation approach. **Results:** The model achieved a 100% trust score, with 20% of the participants reporting full trust and 80% expressing conditional trust, understanding the diagnosis but seeking further clarification. Overall, the participants reported that the integrated explanation format improved their understanding of the model decisions and enhanced their confidence in using AI-assisted diagnosis. **Conclusions:** To our knowledge, this paper is the first to gather the views of medical experts to evaluate the explainability of an AI decision-making model when diagnosing AD. These preliminary findings suggest that explainability plays a key role in building trust and ease of use of AI tools in clinical settings, especially when used by experienced clinicians to support complex diagnoses, such as AD.

## 1. Introduction

Alzheimer’s disease (AD) presents major diagnostic challenges, particularly in its early stages [1,2,3]. Recent developments in artificial intelligence (AI) have led to the development of decision support tools [4,5,6,7]. Some enable doctors to analyze clinical data and medical imaging [2,8,9]. However, the ambiguous nature of many AI models, known as the “black box” problem, limits their use in clinical settings. Explainable AI (XAI) aims to address this limitation by making AI decisions explainable and reliable for end users [10,11,12,13]. Explainability is especially important in clinical contexts [1,14,15], where clinicians must understand and justify diagnoses based on both medical knowledge and patient data [8,13,16,17].

To improve the explainability of diagnostic models for AD, we introduce the Feature-Augmented framework, an AI framework that integrates rule-based explanations with example-based visualizations. This approach combines the strengths of interpreting clinical data with visual signals from MRI scans to provide a comprehensive explanation of the model’s decision-making process. This paper presents the results of a small-scale survey conducted with experienced clinicians to assess the effectiveness of the “Feature-Augmented” approach. This study explores how integrated explanation mechanisms can support clinical decision-making and enhance clinicians’ confidence in AI-based diagnostics.

## 2. Related Work

### 2.1. Background of Explainable Artificial Intelligence (XAI) in Alzheimer’s Diagnosis

Recent years have seen significant development in the use of AI models for the early detection of AD [18,19,20,21], particularly using MRI data and clinical data, such as the Mini-Mental State Examination (MMSE). Despite the remarkable success in improving the accuracy of predictive models, many of these models rely on deep and complex neural networks [22,23,24,25,26,27,28,29,30,31,32,33], whose decisions are difficult to interpret, reinforcing the “black box” problem in sensitive medical systems [20]. For example, some models have relied on processing all brain segments to extract predictive features [26,28,34], which has complicated the output and made it difficult for non-specialized clinicians to track the logic of decision-making [35].

To address the explainability problem, several studies have used XAI tools in AD systems. SHAP techniques are effective at displaying the relative importance of each patient’s clinical characteristics, enabling clinicians to follow the model’s rationale more clearly. Furthermore, Grad-CAM has been utilized with CNN models to analyze MRI images and identify brain regions that affect diagnosis, thereby providing visual interpretations that support model decisions [35]. Some studies [36,37] have built hybrid models to combine outputs and achieve high accuracy. However, interpretation remains challenging when presenting the results to users [17,23]. The use of ensemble learning with parallel learning enables the filtering of irrelevant features and focuses on those most strongly associated with AD [38,39]. This approach ensures that XAI outputs are generated based on more reliable predictions, thereby increasing clinicians’ trust in the model.

However, while these approaches have improved interpretability to some extent, the evaluation of how well clinicians understand and trust XAI outputs remains largely unexplored. To our knowledge, existing XAI studies in Alzheimer’s diagnosis (e.g., those integrating SHAP or Grad-CAM with clinical datasets) have not incorporated formal metrics for trust or usability, highlighting an important area for future work in evaluating explainability effectiveness.

**Figure 1:**
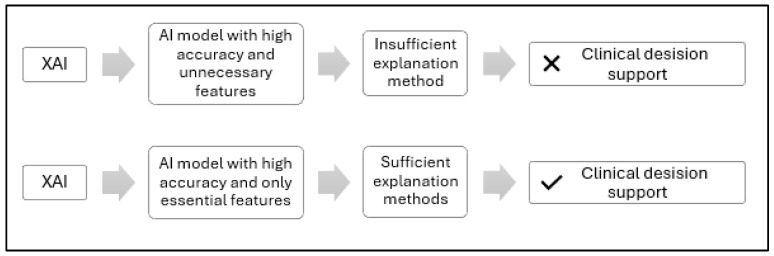
Conceptual comparison of standard and proposed XAI feature extraction approaches for clinical decision support.

Figure 1 is a conceptual illustration comparing two approaches to interpreting XAI-based clinical decisions. The upper section illustrates a typical XAI scenario, where feature extraction involves both essential and non-essential features, often relying on a single model, all MRI slices, or limited clinical data, resulting in insufficient annotation methods and low physician confidence. In contrast, the bottom section represents our proposed Feature-Augmented XAI approach, which uses ensemble learning with parallel learning to retain only the most important features, and applies multiple annotation methods, including SHAP for rule-based feature attribution and Grad-CAM for MRI saliency mapping, to make AI decisions more transparent and actionable for healthcare providers.

### 2.2. Problem Formulation

Despite significant advances in the performance of AI models in diagnosing AD, the “black box” problem remains a major challenge in adopting these models in actual clinical contexts. Most deep learning-based models are highly accurate but lack explainability, which undermines physicians’ confidence in their results and hinders their integration into clinical decisions [24,31,32,40,41].

Previous studies relied on fully processing MRI using all slices; this approach contributed to the complexity of clinical interpretation of the results, as it was not clear whether all slices were of equal importance in diagnosis or not [18]. This leads many current AI methods, such as Grad-CAM [27,29] or LIME [28,34], to highlight areas outside the brain as most important for classification, reducing trust in such AI tools within the medical domain. Moreover, many interpretation tools provide results that are difficult for end users to understand. For example, applying SHAP to MRI data often produces outputs that are challenging to interpret [26]. Current explanation tools are limited to uninterpretable heatmaps generated by Grad-CAM or to numerical values produced by SHAP without a direct connection to the clinical context [30].

Most current studies focus on providing only one explanation using SHAP or Grad-CAM [26,27,28,29,30,34], which is insufficient to build real confidence in the model’s decisions, particularly in an Alzheimer’s diagnosis, a sensitive medical application [14]. While several recent studies have integrated SHAP or Grad-CAM with clinical datasets and MRI, routine deployment within hospital workflows remains rare due to limited trust in the decision-making process and concerns over the reliability of AI-generated explanations.

It is essential to provide multiple, integrated explanations—visual, textual, and numerical—to meet the needs of clinicians, researchers, and practitioners. Such an approach would enhance the model’s interpretability and user confidence, while also supporting local interpretation of individual cases, as clinical decisions are often based on assessing a specific patient’s condition.

Explanation methods in XAI are classified according to several key dimensions that help with choosing the most appropriate tool or methodology based on the type of model and the purpose of the explanation [35]. (1) In terms of scope, explanations can be global, explaining the decision-making mechanism of the model as a whole, or local, focusing on explaining a specific decision for a specific data point. (2) In terms of the implementation method, some methods are applied after training the model (post hoc) to interpret it without modification, while others are integrated into the model structure from the beginning (ante hoc). (3) In terms of the form of explanation, results may be visual (e.g., heat maps), textual (human-readable descriptions), numerical (highlighting the quantitative impact of each variable), or logical (rule-based). (4) In terms of applicability, there are model-agnostic methods usable with any machine learning model and model-specific methods tailored to certain architectures.

**Figure 2:**
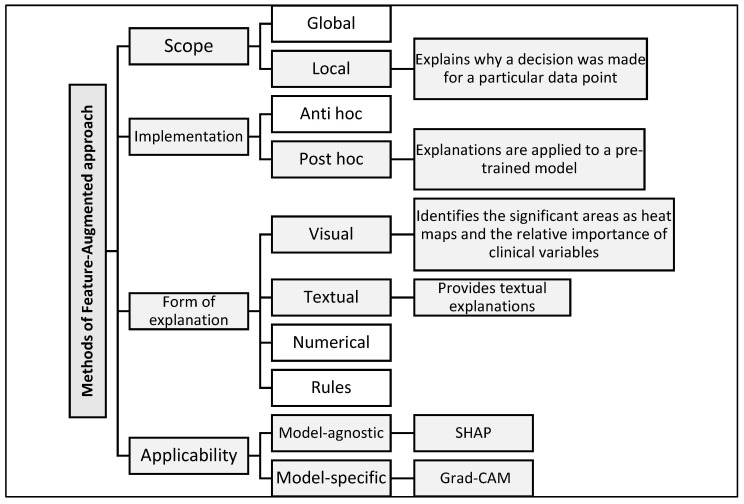
Classification of explanation methods used in the proposed Feature-Augmented approach.

By reviewing [35], our model contributes to addressing several gaps and criticisms raised by this reference. This gap was addressed by developing a Feature-Augmented approach, which combines multiple interpretive tools to present accurate and understandable results for users in real-world clinical settings. Figure 2 illustrates the structure of the proposed Feature-Augmented approach, showing how SHAP-based clinical explanations and Grad-CAM MRI visualizations are combined into a single, integrated interface that enhances both the accuracy and interpretability for clinical decision-making.

The reliance on single explanations, highlighting irrelevant brain regions, and producing outputs disconnected from the clinical context are limitations in current XAI tools for AD diagnosis. These limitations motivated the development of our proposed framework, which integrates parallel ensemble learning with multimodal explanations to enhance the trust and interpretability. The innovation in our study was the exclusive reliance on the mid-slice to reduce the model complexity and enhance the transparency, enabling clinicians to understand model decisions more easily. In addition, clinical data and MRI results were integrated within the parallel ensemble learning model and subsequently employed (SHAP and Grad-CAM) to provide targeted visual and textual interpretations for different categories of users, including doctors and medical students.

## 3. Methods

### 3.1. Objective and Theoretical Foundation of the Feature-Augmented Framework

The main objective of this research is to improve the explainability and usability of AI tools for medical experts in real-world clinical settings. To achieve this, the Feature-Augmented approach is proposed for early AD classification. This framework integrates rule-based interpretations (SHAP) with example-based visual interpretations (Grad-CAM) into a unified system that supports clinical decision-making in crowded or resource-limited environments where specialists may be absent.

The approach combines feature attribution techniques and image saliency maps, enhanced with textual explanations, to help general practitioners and non-specialists identify key clinical and imaging indicators of AD. To ensure accurate interpretation, clinical data are integrated with imaging information from only the middle MRI slices that show the lateral ventricles, as ventricular dilatation is a well-established hallmark of AD progression [42]. Focusing on this slice provides a high signal-to-noise ratio by excluding the less informative upper and lower brain regions, thereby reducing irrelevant activation in the Grad-CAM heatmaps. From a diagnostic perspective, concentrating on the mid-slice allows the model to learn patterns closely aligned with radiological practice, which improves both the classification accuracy and interpretability without requiring full-volume MRI processing [42].

After extracting these relevant features, ensemble learning is applied to emphasize the important attributes and filter out irrelevant ones, ensuring reliable predictions before presenting the interpretation results.

The theoretical formulation of the Feature-Augmented approach relies on three key components: First, a confidence score is provided to reflect the reliability of the prediction. Second, the feature attribution explanation is generated using SHAP values, which quantify the contribution of each clinical feature to the model’s prediction:

(1) $$S_{clinical}\;(x)\;=\;\varphi_{1}\cdot x_{1}\;+\;\varphi_{2}\cdot x_{2}\;+\;\ldots \;+\;\varphi ₙ\cdot xₙ$$

The most important values of SHAP on which the interpretation is based are explained. Third, the visual explanation is provided using Grad-CAM, which highlights important regions in the MRI slice that influenced the model’s decision:

(2) $$S_{image}(I)\;=\;\sum ₖ\;\alpha ₖ\cdot Aₖ$$

This visual map is displayed alongside a textual description of the affected brain regions. Since the MRI was trained on only the middle slice of the brain, which clearly shows the lateral ventricles, Grad-CAM correctly visualizes them as the regions that most influenced the decision, increasing the confidence in the model. Thus, the explanation is reliable and can be used clinically.

Table 1 provides an organized overview of the study phases conducted in this study. Each stage addresses a specific aspect of the proposed enhancement. The table summarizes the research topic, methodology, performance measures, and validation approach taken at each stage.

### 3.2. Feature-Augmented Explainable Artificial Intelligence

**Figure 3:**
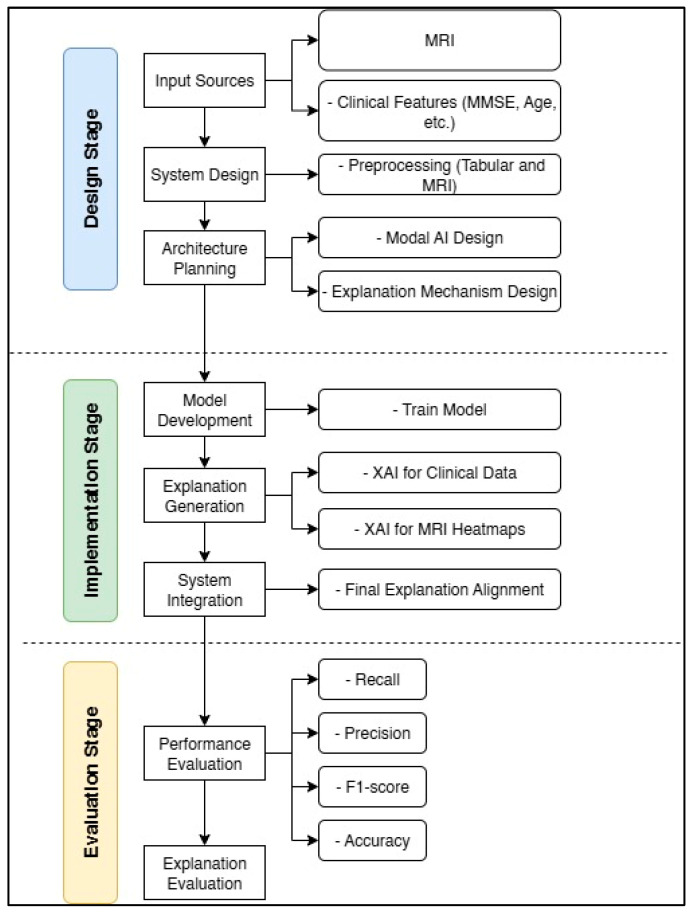
Block diagram of the design, implementation, and evaluation stages for the Feature-Augmented approach in this study.

This section describes the methodology employed in this research, which comprises three main phases: design, implementation, and evaluation. These stages are summarized in Figure 3, which displays a schematic diagram of the general framework.

#### 3.2.1. Model Architecture and Ensemble Design

As detailed in our previous work [42], clinical data are preprocessed by encoding categorical variables, creating additional ratio features, and scaling numeric values. MRIs are processed separately by extracting deep features using a pretrained ResNet50 model, followed by normalization. After preprocessing, the clinical and MRI features are horizontally concatenated to form a combined feature vector representing both data types for each patient. This fusion allows base classifiers to learn from the integrated information in a unified input space. Prediction probabilities from each base model are then combined, and a logistic regression meta-learner learns to balance them for improved accuracy. To preserve interpretability, explanations are generated separately for each data modality (SHAP for clinical features and Grad-CAM for MRI images), ensuring that combining the outputs does not reduce the clarity of the explanations presented to the end user.

We used an ensemble learning strategy [38,39,43,44,45,46,47]; ensemble learning allows each model within our selected set to focus on specific, high-impact features of both clinical and MRI data to produce clearer interpretations.

Our approach was based on four basic classifiers: Random Forest (RF), Extreme Gradient Boosting (XGB), Support Vector Machine (SVM), and Gradient Boosting (GB). The RF classifier aggregates predictions from multiple decision trees using majority voting. The final prediction  ŷ  is given by majority voting [45,48]:

(3) $$\;ŷ\;=\;mode(y_{1},\;y_{2},\;\ldots ,\;yₙ)\;\;$$

where yₙ is the class predicted by the $i^{th}$ tree.

XGB sequentially builds trees and minimizes a regularized objective function [49]:

(4) $$L\left(t\right)\approx \sum_{\left\{i=1\right\}}^{\left\{N\right\}}\left[gᵢ\cdot fₜ(xᵢ)\;+\;(1/2)\cdot hᵢ\cdot {fₜ}^{2}(xᵢ)]\;+\;\Omega (fₜ\right)$$

SVMs are effective for well-separated data. The binary decision function is defined as follows [50]:

(5) $$f(x)=sign(\sum_{\left\{i=1\right\}}^{\left\{T\right\}}\alpha ᵢ\;yᵢ\;K(xᵢ,\;x)\;+\;b)$$

where αᵢ are Lagrange multipliers, yᵢ ∈ {±1}, k is the kernel function, and b is the bias.

GB iteratively fits new learners to the negative gradient of the loss function [49]:

(6) $$g_{t\left(x\right)}\;=\;E_{y}\left[\;\left.\begin{array}{c} \frac{\partial \psi \left(y,\;f\left(x\right)\right)}{\partial f\left(x\right)} \end{array}\right|x\;\right]\;\;\;\;where\;\;\;\;f\left(x\right)=\;f^{\left\{t-1\right\}\left(x\right)}$$

Ensemble learning works by aggregating predictions from baseline models, reducing unnecessary features, and enhancing the model explainability. Our meta-model learns optimal weights to combine predictions through five-fold cross-validation. It performed the final classification into three classes: Alzheimer’s disease (AD), mild cognitive impairment (MCI), and normal cognitive impairment (CN). This architecture improves the performance by leveraging tabular and image-based features while maintaining explainability. The equation of the loss function used to train the meta-learner in our ensemble model is as follows [43]:

(7) $$L\left(\theta \right)=\;-\;\;\sum_{\left\{i=1\right\}}^{\left\{N\right\}}[\;yᵢ\;log(ŷᵢ)\;+\;(1\;-\;yᵢ)\;log(1\;-\;ŷᵢ)\;]\;+\;{\lambda \|\theta \|}^{2}$$

Binary cross-entropy (logarithmic loss) measures how closely the predicted probabilities ŷᵢ match the correct binary labels yᵢ. The L2 regularization threshold is controlled by the parameter λ, which penalizes large weights in the model to reduce overfitting. This combination helps the model make accurate predictions and achieve better generalization.

#### 3.2.2. Explainability Framework

We adopted a dual interpretation strategy using two integrated XAI methods. For the clinical model, SHAP (SHapley Additive Interpretation) was used to generate feature importance values. As for the CNN-based MRI model, Grad-CAM (Gradient-Weighted Class Activation Mapping) was used to generate heatmaps indicating which image regions contribute to the model’s prediction.

This dual framework addresses concerns about ambiguity resulting from using multiple XAI tools on the same pattern of data. By assigning SHAP to clinical data and Grad-CAM to images, we avoided interpretive inconsistencies and enhanced the clarity of the interpretations.

## 4. Experimental Results and Discussion

This dual framework addresses concerns about ambiguity resulting from using multiple XAI tools on the same pattern of data. By assigning SHAP to clinical data and Grad-CAM to images, we avoided interpretive inconsistencies and enhanced the clarity of interpretations.

### 4.1. Data Processing

For the MRI data, we specifically selected medial slices that prominently showed the lateral ventricles since these regions are clinically associated with brain atrophy in AD. This approach is consistent with recommendations in previous references to focus on regions with clearer underlying anatomical facts to obtain more interpretable output.

**Figure 4:**
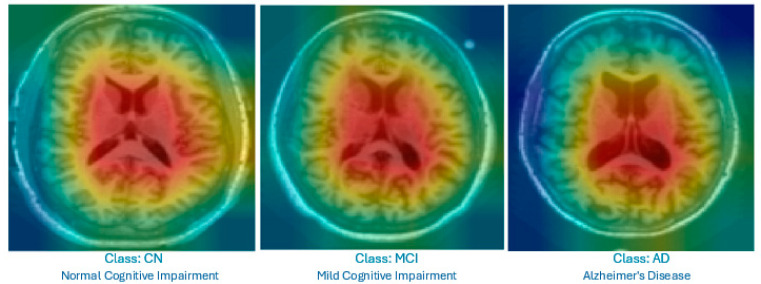
Grad-CAM heatmap showing the highlighted lateral ventricles in a mid-slice MRI; this was produced by the proposed Feature-Augmented XAI approach to explain how the diagnosis of AD was made.

Figure 4 displays the Grad-CAM heatmaps produced by the proposed improved model for each category. The visualizations highlight the lateral ventricles, which are the primary focus areas used by the model for decision-making. These regions correspond to known clinical indicators of AD progression.

Clinical data were also used, with clinical characteristics selected based on their proven relationship in the medical literature with the stages of AD progression, along with dilatation of the lateral ventricles on MRI images. Clinical features included demographic factors, such as age and gender, given their direct impact on the risk of developing AD, as well as years of education as an indicator of cognitive reserve, which may influence resistance to cognitive decline. The Mini-Mental State Examination (MMSE) scores, a primary standardized tool for assessing cognitive function, and the Clinical Dementia Score (CDR), which helps classify the severity of the condition, were also included. In addition, other indicators were used, such as delayed memory performance and the level of independence in daily activities, as represented by the FAQ scale.

All MRIs were scaled and normalized, and the clinical data underwent processing steps, such as missing value imputation and normalization. The final dataset was weighted to address problems of class imbalance observed in previous studies. The final model, trained on clinical data and MRI mid-slices, achieved 99.00% accuracy. Moreover, the use of ensemble learning significantly improved the computational efficiency and reduced the processing time. After the high-performance prediction phase, the next crucial step is the explanation process, where the XAI module is activated to provide clear and explainable justifications for every decision the model makes.

The full implementation code used for this study is available at https://github.com/F-H5/diagnosis_explanation_module (accessed on 15 May 2025).

### 4.2. Model Configuration and XAI Integration

The proposed model accepts two main types of input: (1) clinical features, including demographic and cognitive test results, and (2) intermediate MRI slices, specifically selected to capture the lateral ventricles, which are known to show structural changes in AD patients.

To ensure transparency, each data type is associated with a custom annotation method:

- SHAP is applied to clinical data. This model quantifies the contribution of each feature to the final prediction and presents the results in graphical and textual form, making them easier for clinicians to understand.
- Grad-CAM is used for MRI images. It produces heat maps superimposed on the original slices to highlight the brain regions that influenced the classification, helping non-radiologists visualize relevant anatomical patterns.

The outputs of the Feature-Augmented XAI approach consist of three key components: (1) A confidence score is provided to reflect the reliability of the prediction. (2) The feature attribution explanation is generated using SHAP values, which quantify the contribution of each clinical feature to the model’s prediction. The most important values of SHAP on which the interpretation is based are explained. (3) The visual explanation is provided using Grad-CAM, which highlights important regions in the MRI slice that influenced the model’s decision. This visual map is displayed alongside a textual description of the affected brain regions. Since the MRI data were trained on only the middle slice of the brain, which clearly shows the lateral ventricles, Grad-CAM correctly visualizes them as the regions that most influenced the decision. This increases confidence in the model and supports clinical use.

### 4.3. Performance Evaluation

#### 4.3.1. Questionnaire Design and Expert Involvement

To evaluate the clarity, usefulness, and reliability of the explanations generated by the Feature-Based Explainable, a case study was designed and conducted [51]. The study targeted healthcare professionals to assess how different user groups perceive and interact with the outcomes of the explanations. Participants were shown model predictions, SHAP-based feature importance, Grad-CAM visualizations, and accompanying textual explanations, and then asked to provide feedback through a structured questionnaire assessing the interpretability, confidence, and overall satisfaction.

A total of five participants specialized in the field of AD, each with 6 to 10 years of experience, completed the questionnaire. Each case was accompanied by one of the following:

  i. SHAP-based clinical explanation only: Highlighting key clinical features.
 ii. Grad-CAM heatmaps only: Showing important brain regions in the MRI.
iii. Feature-Augmented XAI explanation: Combining SHAP-based features, Grad-CAM heatmaps, and textual explanations.

While the expert validation in this study provides valuable insights, it is important to recognize the limitations imposed by the small sample size (*n* = 5). Since this study was designed as a pilot study, the main objective was to explore the feasibility and initial impressions of the proposed Feature-Augmented approach rather than to achieve statistical generalizability. The limited number of participants is primarily due to time constraints and the availability of experienced physicians willing to participate, as noted in similar research challenges [14]. Therefore, the results should be interpreted with caution, as they may not fully represent the views of the wider medical community.

Evaluation criteria: These criteria represent the evaluation instrument used to assess participants’ perceptions of the XAI outputs. The participants were asked to rate the model’s interpretability and usefulness based on these four dimensions:

  i. Clarity: How easy the interpretations were to understand.
 ii. Clinical relevance: The extent to which interpretations align with known biomarkers of AD.
iii. Decision support: The extent to which interpretations contribute to clinical decision-making.
iv. Confidence in AI: Participants’ degree of confidence in AI-assisted diagnosis after reviewing the interpretations.

A Likert scale (1–5) was used, and qualitative feedback was collected to gain deeper insights into participants’ perspectives.

#### 4.3.2. Expert Validation Results

The main performance indicator employed in this phase was the trust score [52], which quantifies expert confidence in the model’s explanations. The model achieved a 100% trust score, with 20% of participants reporting full trust and 80% expressing conditional trust. Feedback from Alzheimer’s specialists confirmed its potential to support clinical decision-making, especially in contexts with limited access to specialists.

The proposed Feature-Augmented XAI demonstrated improved interpretability and clinical relevance by integrating SHAP for clinical features and Grad-CAM for MRI imaging, providing both visual evidence and quantitative feature contributions.

**Figure 5:**
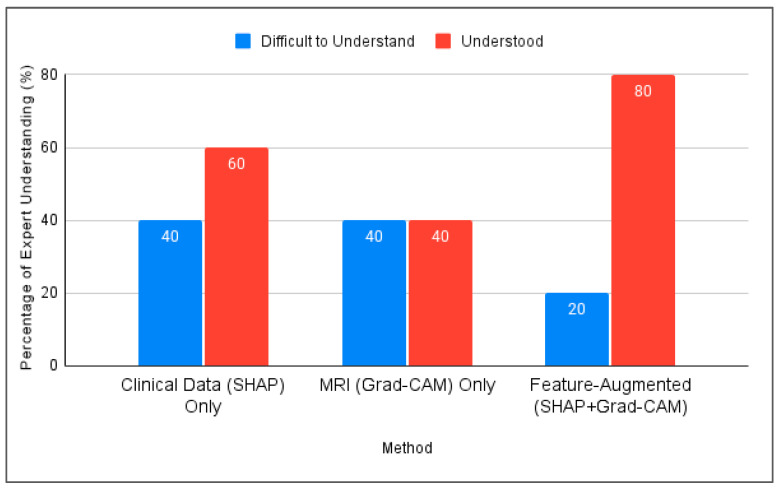
Comparison of expert understanding between SHAP, Grad-CAM, and the proposed Feature-Augmented approach in this study.

**Figure 6:**
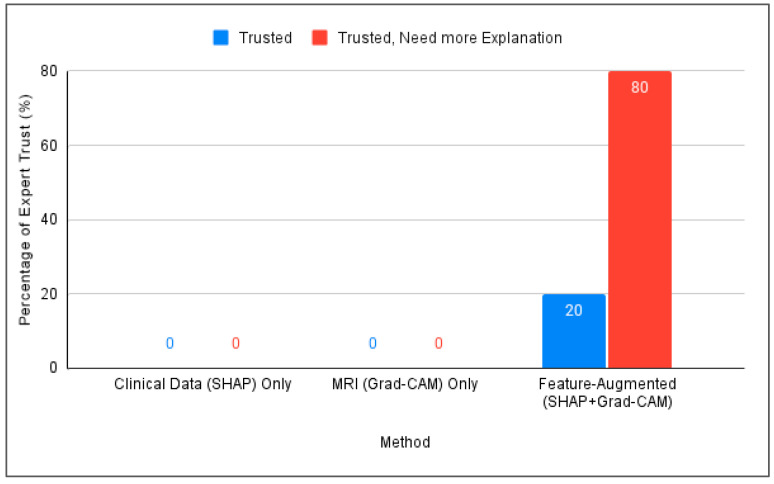
Comparison of expert trust levels across SHAP-Only, Grad-CAM-Only, and proposed Feature-Augmented explainability methods.

Figure 5 compares the three interpretation approaches: SHAP alone, Grad-CAM alone, and Feature-Augmented XAI. SHAP alone was found difficult to understand without additional explanation. Grad-CAM was visually understandable but insufficient for diagnostic confidence, while Feature-Augmented XAI improved both understanding and trust (Figure 6).

Trust means that the specialist can understand the interpretation and trust the diagnosis based on it. To calculate the trust score in this study, a direct measurement approach was adopted based on the experts’ responses. This method is consistent with the approach used by [52], who calculated the trust score as the average of self-reported confidence levels using a Likert scale ranging from 1 to 5. The formula for the direct trust score is defined as follows [52]:

(8) $$Trust\;Score=\frac{Number\;of\;participants\;who\;trust\;the\;model}{Total\;number\;of\;participants}\times \;100\%$$

Based on the participants’ responses, since all participants indicated trust, either directly or conditionally, the overall trust score for Feature-Augmented XAI is considered 100%, with a note that most users desire enhanced interpretability.

As shown in Table 2, all experts had more than ten years of experience and demonstrated familiarity with AD diagnosis and XAI, with varying degrees of confidence in the proposed model.

Key findings from the expert evaluations include the following:

- All five experts agreed that integrating clinical, MMSE, and MRI data improves the diagnostic accuracy.
- Four out of five agreed that AI models like ours help detect subtle disease patterns that are not easily visible through human interpretation.
- All experts rated SHAP and Grad-CAM explanations as either “understandable” or “very understandable” and described them as useful for gaining insight into the model decisions.
- Three out of five experts suggested that the textual explanations could be made clearer, especially when intended for non-expert users.
- All five participants emphasized that such explainability tools should be used to support, not replace, human physicians and showed strong support for integrating these models into future clinical practice.

As noted in the comments, experts generally found the model explanations clear and helpful; however, some have suggested further simplifying the textual output for non-expert audiences. Suggested improvements include using simpler medical terminology, shorter sentences, and adjusting the level of detail to suit the needs of different clinical populations to ensure explanations are clear and accessible to a wider range of users.

#### 4.3.3. Expert Reflections on Explanation and Clinical Relevance

In addition to the quantitative responses, experts provided open feedback. They confirmed that the visual interpretations—those focusing on the lateral ventricles—matched their clinical expectations for diagnosing AD. One expert noted that combining textual justifications with visual output “bridges the gap for non-specialists and supports clinical training.” Another expert highlighted the utility of SHAP outputs in “understanding the contribution of MMSE subitems, especially in borderline MCI cases.” A common theme among the responses was an appreciation for the clear segmentation between interpretations of clinical data and interpretations of imaging, which facilitated a more intuitive understanding of the decision-making process.

One expert raised a valuable point regarding the specificity of cognitive assessments, noting that “cognitive tests may be abnormal in depression or other psychiatric conditions.” This highlights the importance of considering differential diagnoses and reinforces the need for multimodal approaches that combine imaging and clinical data to reduce the potential for misclassification. Moreover, the experts agreed that these interpretive approaches are critical not only for enhancing confidence in AI models but also for promoting their adoption in multidisciplinary medical teams. These qualitative insights demonstrate the importance and usability of the Feature-Augmented XAI approach in the real world.

### 4.4. Discussion

In our previous study, we developed an improved Feature-Augmented XAI framework for AD detection, balancing high accuracy and ease of interpretation [42]. Our proposed model addresses limitations highlighted in prior works [15,16,35,53,54,55] by involving experts in validation, interpreting both clinical and imaging data, and avoiding overlap between explanation tools.

#### Addressing XAI Challenges Identified in Prior Work

Based on the limitations highlighted in [4,5,35], our model introduces several solutions that directly address existing gaps in XAI applications for AD diagnosis:

1. Involvement of medical experts: Specialists with over 10 years of experience evaluated the outputs through a structured questionnaire. The model achieved a 100% trust score, confirming improved confidence in AI decisions.
2. Integrating multiple modalities: SHAP was applied to clinical data and Grad-CAM to MRI slices, ensuring comprehensive modality-specific interpretations.
3. Reducing ambiguity from multiple XAI tools: Using several explainability frameworks on the same data can lead to contradictory outputs [35]. Our model avoids this issue by assigning SHAP exclusively to clinical data and Grad-CAM to image data, preventing interpretive overlap and maintaining clarity.
4. Focusing on specific MRI slices to enhance explainability: Our model restricts analysis to middle slices that show the lateral ventricles, which are well-known indicators of AD-related atrophy. This strategy enhances the explainability and clinical relevance of the visual output.
5. Formulating tailored explanations: Textual interpretations were adapted for physicians and medical students to improve the clarity and trust across user groups.

In addition to these advancements, it is important to consider how the mode of presenting explanations, whether SHAP-only, Grad-CAM-only, or the integrated Feature-Augmented approach, impacts clinicians’ cognitive load and decision-making speed, especially in busy clinical settings. While the current study focuses on accuracy and interpretability, future evaluations should incorporate usability testing to assess how sequential versus combined presentation of explanations affects physicians’ comprehension, mental effort, and diagnostic efficiency. Optimizing the delivery of explanations to minimize cognitive load has the potential to enhance user experience and facilitate wider adoption of the model in real-world clinical practice.

## 5. Conclusions

By integrating multi-modal data and relevant clinical characteristics, the Feature-Augmented approach addresses the “black box” problem and helps non-specialists make informed diagnostic decisions. The Feature-Augmented approach successfully achieved a balance between the model accuracy and explainability, an achievement that distinguishes our work from previous studies, which often sacrifice explainability for high accuracy or compromise accuracy to obtain clearer explanations. The Feature-Augmented approach lays the foundation for future developments in transparent AI in healthcare.

Expert feedback confirmed the transparency of the Feature-Augmented explanations, with a 100% trust score indicating strong confidence in the model’s decisions. The provided explanations were found to enhance understanding of the model predictions, facilitate cross-validation with medical knowledge, and increase confidence in the model decisions. The use of regions showing the lateral ventricles in the middle MRI slices, a key marker of AD progression, improved the agreement between Grad-CAM maps and known clinical markers of AD.

As a result, the key outputs of the Feature-Augmented approach were achieved: (1) enhanced explanation quality, (2) reliable clinical decision support, and (3) clear justification. Furthermore, textual explanations tailored to different audience categories have been praised for improving the explainability across user types.

### Future Work

It is important to acknowledge that this study has some limitations, such as the small number of experts involved in the validation phase and reliance on a single data source; these factors affect the generalizability of the results.

Future work aims to incorporate data collected from local populations to validate the tool’s performance across diverse demographic and genetic backgrounds, thereby ensuring the broader generalizability of the results.

Moreover, in future work, multiple models could be trained, each focusing on a single region of interest for AD diagnosis on MRIs. These models could then be combined into a single model that offers multiple interpretations, with each one specific to the model responsible for the training region, and displays textual information for each region to support decision-making.

In addition, future development will explore ways to simplify the model’s textual explanations by using more accessible medical terminology and shorter sentences to meet the needs of different medical audiences, including non-specialists and medical students.

Further evaluations will include a broader range of healthcare professionals, such as general practitioners, nurses, and neurologists, as well as participants from diverse clinical backgrounds, to improve the generalizability and robustness of the conclusions and evaluate the tool’s performance across various clinical aspects and specialties. Future works will compare the Feature-Augmented approach with other XAI approaches using standardized explainability metrics and user satisfaction surveys to position it within the broader landscape of XAI in medicine.

Future work could also focus on deploying this approach within portable diagnostic devices to use in neurology departments and clinical decision support systems. Through these approaches, the Feature-Augmented approach aims to develop into a robust and widely applicable solution that bridges the gap between high-performance AI models and real-world clinical needs, particularly in resource-limited or high-stress settings.

## Figures and Tables

**Table 1:** Overview of the research phases, methodology, and evaluation for the Feature-Augmented approach in this study.

Phase	Subject Matter	Methodology	Measure of Performance	Result Validation
Phase 1	Enhanced XAI (Feature-Augmented Design)	Develop an ensemble meta-model using mid-slice MRI and clinical data; apply SHAP and Grad-CAM for explainability	Accuracy, recall, precision, and F1-score	Compared against standard XAI models; validated using ADNI and OASIS datasets
Phase 2	Integration of Tabular and Image Features	Extract predefined clinical features (e.g., MMSE, age); train a hybrid model with SHAP for interpretation	Explanation coverage and clinical explainability	Measured improvements in classification, accuracy, and explanation quality
Phase 3	Limitations of Current XAI Techniques	Literature gap analysis; compare SHAP-only and Grad-CAM-only models; evaluate enhanced model	Usability score, explanation clarity, and time to understand	Theoretical and practical comparison; discuss model-agnostic vs. model-specific XAI limitations
Phase 4	Evaluation of Explanation Quality and Performance	Collect user and expert feedback via surveys to assess clarity, usefulness, and trust in explanations	Trust, clarity, and usefulness	Validated through structured feedback from experts

**Table 2:** Summary of expert participants’ background and confidence in XAI.

Expert	Years of Experience	Familiarity with Alzheimer’s Diagnosis	Familiarity with XAI in Medicine	Confidence in XAI
Person 1	More than 10 years	Excellent knowledge	Good	Strongly Agree
Person 2	More than 10 years	Excellent knowledge	Moderate	Agree
Person 3	More than 10 years	Excellent knowledge	Excellent	Strongly Agree
Person 4	6–10 years	Good knowledge	Moderate	Agree
Person 5	6–10 years	Good knowledge	Moderate	Neutral

## Data Availability

The source code used to preprocess the data, train the models, and generate the diagnostic explanations is publicly available at the following GitHub repository (Python 3.10): https://github.com/F-H5/diagnosis_explanation_module (accessed on 15 May 2025). The data used in this study were collected through a structured survey of five experts. The survey responses were used solely for research purposes. Due to privacy and ethical considerations, the raw survey data is not publicly available. However, aggregated data and analysis results are available upon reasonable requests from the corresponding author.
